# Deguelin Inhibits the Migration and Invasion of U-2 OS Human Osteosarcoma Cells via the Inhibition of Matrix Metalloproteinase-2/-9 *in Vitro*

**DOI:** 10.3390/molecules191016588

**Published:** 2014-10-15

**Authors:** Hung-Sheng Shang, Jin-Biou Chang, Ju-Hwa Lin, Jing-Pin Lin, Shu-Chun Hsu, Chi-Ming Liu, Jia-You Liu, Ping-Ping Wu, Hsu-Feng Lu, Man-Kuan Au, Jing-Gung Chung

**Affiliations:** 1 Department of Pathology, National Defense Medical Center, Division of Clinical Pathology, Tri-Service General Hospital, Taipei 114, Taiwan; 2 Department of Medical Laboratory Science and Biotechnology, Yuanpei University, Hsinchu 300, Taiwan; 3 Department of Biological Science and Technology, China Medical University, No 91, Hsueh-Shih Road, Taichung 404, Taiwan; 4 School of Chinese Medicine, China Medical University, Taichung 404, Taiwan; 5 Department of Research and Education, Cheng-Hsin General Hospital, No.45, Cheng Hsin St., Pai-Tou, Taipei 112, Taiwan; 6 Department of Clinical Pathology, Cheng-Hsin General Hospital, Taipei 112, Taiwan; 7 School of Pharmacy, China Medical University, Taichung 404, Taiwan; 8 Department of Restaurant, Hotel and Institutional Management, Fu-Jen Catholic University, New Taipei City 242, Taiwan; 9 Department of Orthopedic Surgery, Cheng-Hsin General Hospital, Taipei 112, Taiwan; 10 Department of Biotechnology, Asia University, Taichung 413, Taiwan

**Keywords:** deguelin, U-2OS human osteosarcoma cells, migration, invasion

## Abstract

Osteosarcoma is the most common malignant primary bone tumor in children and young adults and lung metastasis is the main cause of death in those patients. Deguelin, a naturally occurring rotenoid, is known to be an Akt inhibitor and to exhibit cytotoxic effects, including antiproliferative and anticarcinogenic activities, in several cancers. In the present study, we determined if deguelin would inhibit migration and invasion in U-2 OS human osteosarcoma cells. Deguelin significantly inhibited migration and invasion of U-2 OS human osteosarcoma cells which was associated with a reduction of activities of matrix metalloproteinases-2 (MMP-2) and matrix metalloproteinases-9 (MMP-9). Furthermore, results from western blotting indicated that deguelin decreased the cell proliferation and cell growth-associated protein levels, such as SOS1, PKC, Ras, PI3K, p-AKT(Ser473), IRE-1α, MEKK3, iNOS, COX2, p-ERK1/2, p-JNK1/2, p-p38; the cell motility and focal adhesion-associated protein levels, such as Rho A, FAK, ROCK-1; the invasion-associated protein levels, such as TIMP1, uPA, MMP-2. MMP-9, MMP-13, MMP-1 and VEGF in U-2 OS cells. Confocal microscopy revealed that deguelin reduced NF-κB p65, Rho A and ROCK-1 protein levels in cytosol. MMP-7, MMP-9 and Rho A mRNA levels were suppressed by deguelin. These *in vitro* results provide evidence that deguelin may have potential as a novel anti-cancer agent for the treatment of osteosarcoma and provides the rationale for *in vivo* studies in animal models.

## 1. Introduction

Osteosarcoma is the most common malignant bone tumor found in children and adolescents [1,2,3]. It affects the distal long bones *via* the formation of neoplastic bone tissue [4]. The main cause of death of osteosarcoma patients is lung metastasis [5,6]. The five-year survival rate of patients with osteosarcoma are no greater than 30% after the detection of lung metastasis and lung has some of the most common migration tissues for osteosarcoma and this leads to death within 6 months to one year [7]. Currently, conventional treatments such as surgical resection, chemotherapy, and radiation, or combinations of chemotherapy and radiation are not satisfactory. An effective treatment for osteosarcomas is needed. 

Cancer cell invasion and metastasis lead to a poor prognosis and are a therapeutic challenge [8]. These cellular processes are complex involving activation/degradation of extra-cellular matrix (ECM) and the break-down of ECM by proteinases [9,10,11,12]. Matrix metalloproteinases (MMPs) are a family of ECM degrading proteinases which play an important role in cell invasion and metastasis involving ECM degradation [5,13]. There has been great interest in finding effective MMP inhibitors for the treatment of osteosarcoma [14,15,16].

Deguelin is a rotenoid, derived from several plant species, including *Mondulea sericea*. It has been reported to reduce the *in vivo* incidence of chemically induced-tumors in animal models of lung [17], skin [18] and mammary [18,19] tumors. Deguelin suppressed the formation of cazoxymethane (carcinogen)-induced aberrant crypt foci in mouse colon [20]. Deguelin is a potent inhibitor of ornithine decarboxylase (ODC) activity [21] and it also can trigger inhibition of the PI3K/Akt pathway [22,23] and down-regulation of cyclooxygenase-2 [22]. It was reported that deguelin inhibits the NF-κB activation pathway, which may explain its role in the suppression of carcinogenesis and cellular proliferation [24]. Degulin had been reported to affect different types of human cancer cells but there is no available information on its effects on migration and invasion. In the present study, we investigated the effects of deguelin on the migration and invasion in human bone cancer U-2 OS cells. Deguelin inhibited the migration and invasion of U-2 OS cells by down regulation of MMP-2 and -9. 

## 2. Results and Discussion

### 2.1. Deguelin Reduces the Percentage of Viable U2-OS Human Osteosarcoma Cells

**Figure 1:**
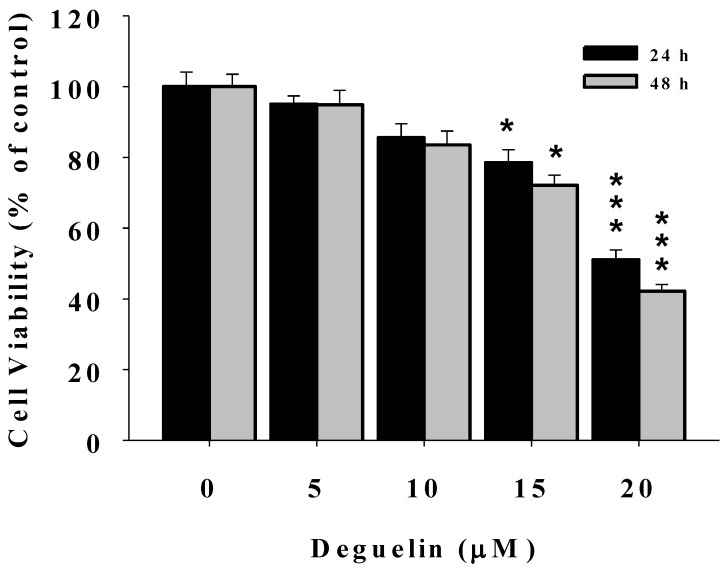
Deguelin affects the percentage of viable U-2 OS human osteosacroma cells. U2-OS cells (2 × 10^5^ cells/well) were incubated with 0, 5, 10, 15, and 20 μM of deguelin for 24 h and 48 h. Cell were harvested to determine the percentage of viable cells by flow cytometry as described in the Experimental Section. * *p* < 0.05, *** *p* < 0.001, significant difference between deguelin-treated groups and the control as analyzed by Student’s *t* test.

After U-2 OS cells were treated with 0, 5, 10, 15 and 20 μM of deguelin for 24 and 48 h, cells were harvested and the percentage of viable cells were determined. Figure 1 shows that deguelin significantly decreased the percentage of viable cells at 15 and 20 μM. Deguelin concentrations of 10 and 15 μM were used for all experiments.

### 2.2. Deguelin Suppresses the Migration of U-2 OS Cells in Vitro

**Figure 2:**
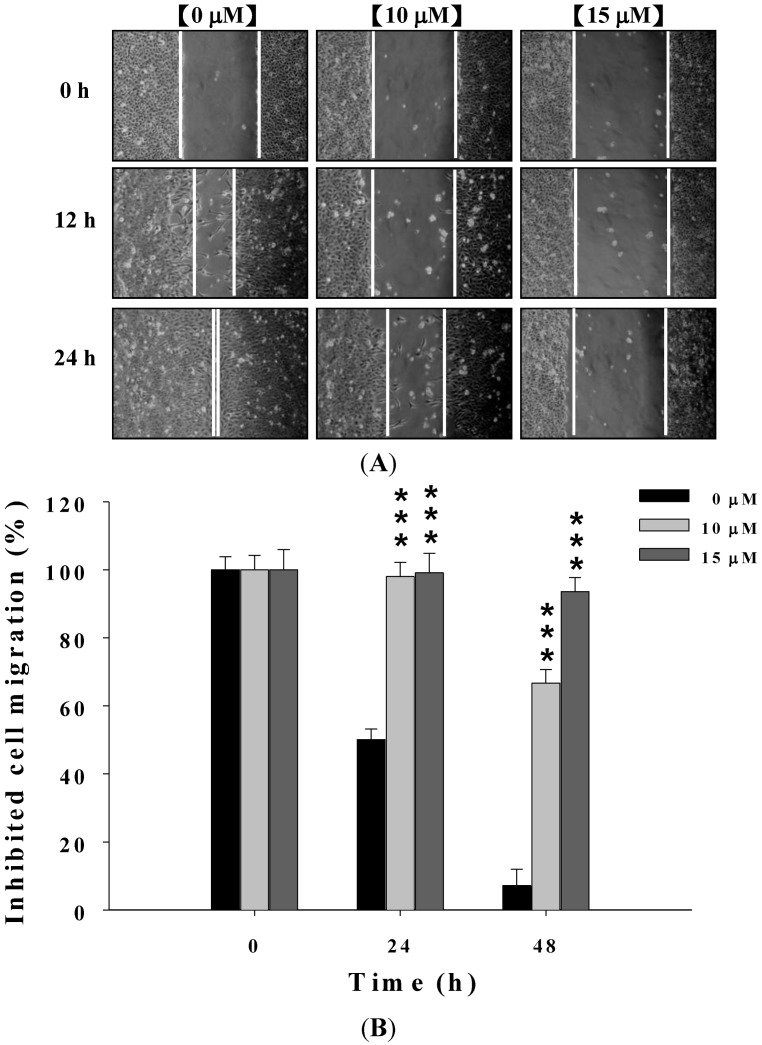
Deguelin affecting the migration of U-2 OS cells examined by wound healing assay. Cells (2 × 10^5^ cells/well) were placed on the dish for 24 h before a wound was produced by scraping confluent cell layers with a pipette tip. Deguelin was added to the well at the final concentration (0, 10 and 15 μM) then incubation for 0, 12 and 24 h (**A**). Some representative photographs of migrating treated and untreated cells are presented. The migrated cells in the five random fields after exposure for 0, 12, 24 h were counted to quantify, and data was expressed as mean ± S.D. *** *p* < 0.001 significant difference between deguelin-treated groups and the untreated groups as analyzed by Student’s *t* test (**B**).

Effects of deguelin on migration and invasion of U-2 OS cells was determined using a wound healing assay. U-2 OS cells were exposed to 0, 10, and 15 μM of deguelin, after which, as seen in Figure 2, deguelin inhibited the migration of U-2 OS cells in a dose- and time-dependent manner. 

### 2.3. Deguelin Inhibited the Migration and Invasion of U2-OS Cells in Vitro

In order to confirm the effect of deguelin on migration and invasion of U-2 OS cells, cells were treated with 0, 10 and 15 μM of deguelin for 24 and 48 h and seeded on Millicell chambers with uncoated (for migration) or matrigel-coated (for invasion) filters.

**Figure 3:**
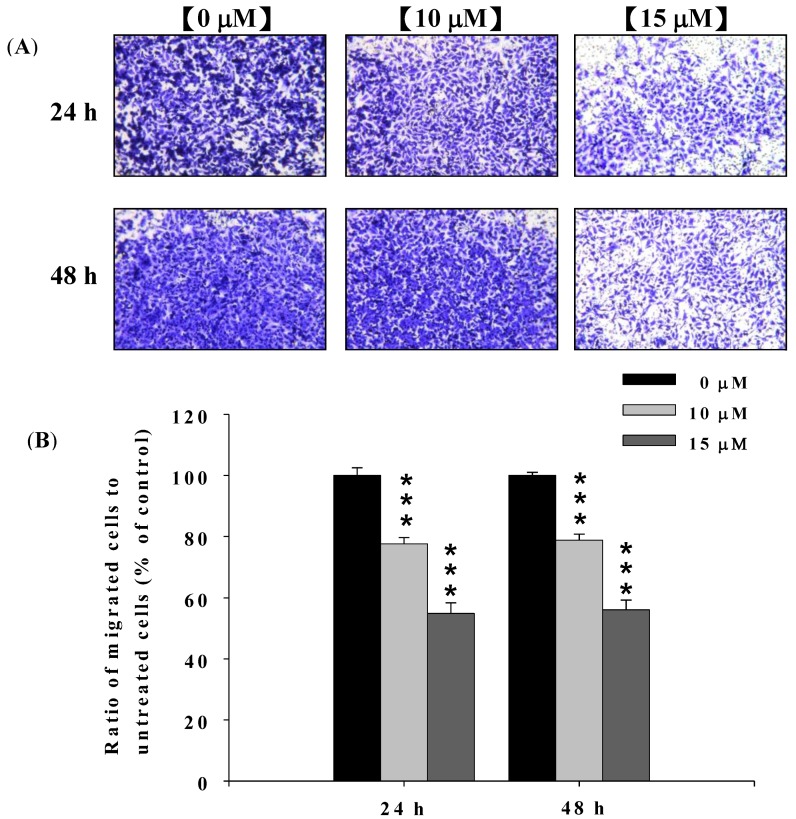
Deguelin suppressed the migration of U-2 OS cells *in vitro*. U-2 OS cells (5 × 10^4^ cells/well) were incubated with 0, 10 and 15 μM of deguelin for 24 and 48 h then penetrated through to the lower surface of the filter and were stained with crystal violet and were photographed under a light microscope at 200×. Quantification of cells in the lower chambers was performed by counting cells at 200×. *** *p* < 0.001 significant difference between deguelin-treated groups and the untreated groups as analyzed by Student’s *t* test. Cells were stained with crystal violet and then were examined and photographed under a light microscope at 200× (**A**); The quantification of cells from each treatment in the lower chambers was counted at 200× (**B**).

**Figure 4:**
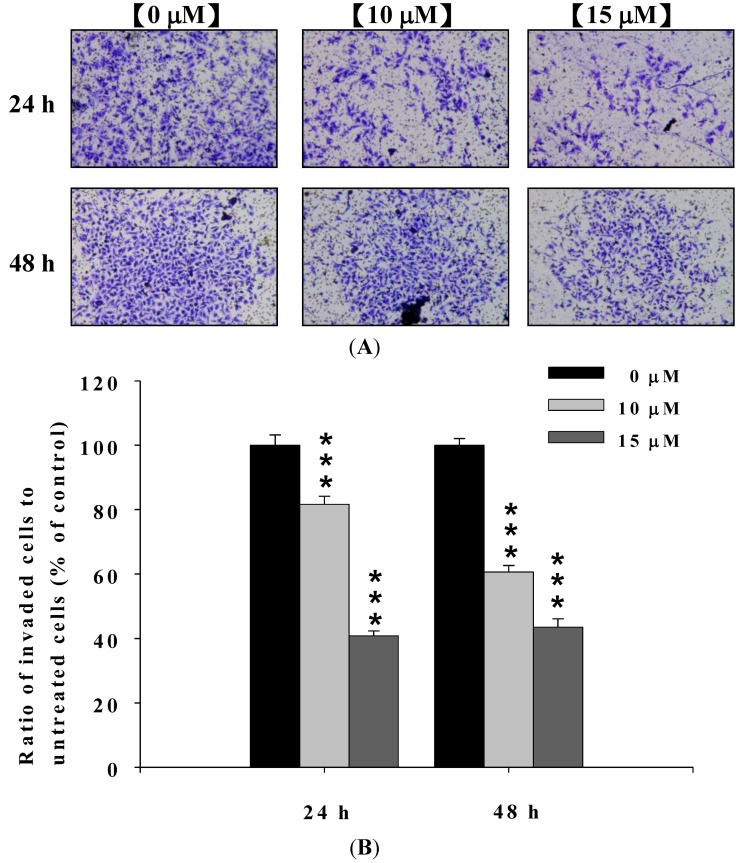
Deguelin suppressed the invasion of U-2 OS cells *in vitro*. U-2 OS cells (5 × 10^4^ cells/well) were incubated with 0, 10 and 15 μM of deguelin then penetrated through with the matrigel to the lower surface of the filter were stained with crystal violet and were photographed under a light microscope at 200×. Quantification of cells in the lower chambers was performed by counting cells at 200×. *** *p* < 0.001, significant difference between deguelin-treated groups and untreated-groups as analyzed by Student’s *t* test. Cells were stained with crystal violet and then were examined and photographed under a light microscope at 200× (**A**); The quantification of cells from each treatment in the lower chambers was counted at 200× (**B**).

At the end of incubation, cell migration activity and invasive potential of U-2 OS cells were measured, photographed and results are shown in Figure 3A,B and Figure 4A,B. Figure 3A,B indicated that deguelin significantly inhibited migration of U-2 OS cells *in vitro* and this effect was dose-dependent. Figure 4A,B indicated that deguelin significantly inhibited the invasion of U-2 OS cells and these effects were dose-dependent.

### 2.4. Deguelin Inhibited the Matrix Metalloproteinases-2 and -9 Activities in U-2 OS Cells

**Figure 5:**
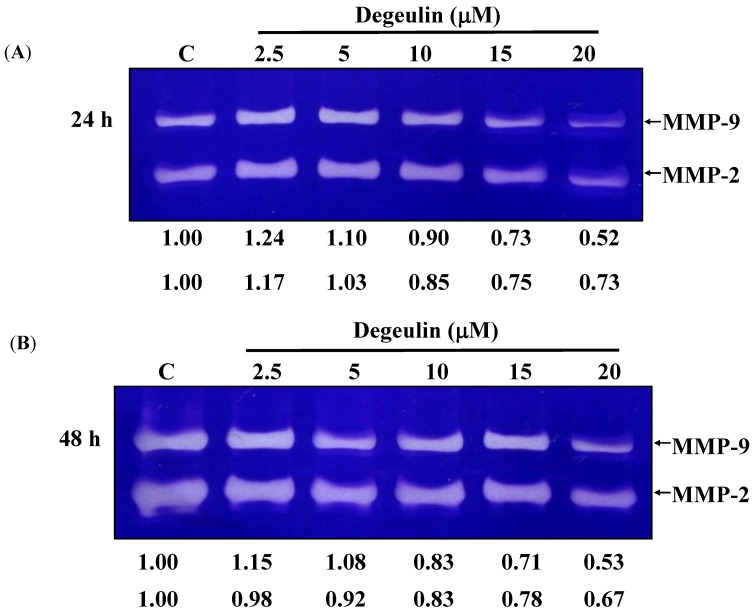
Deguelin affects the activities of MMP-2 and -9 activities in U-2 OS cells. U-2 OS cells (5 × 10^4^ cells/well) were incubated with 0, 2.5, 5, 10, 15 and 20 μM of deguelin for 24 (**A**) and 48 h (**B**) then to detect the activity of secreted MMP-2 and MMP-9 using conditioned medium from U-2 OS cells. The different activity of MMP-2 and MMP-9 were determined by densitometric analysis and results are expressed as a percentage of the control (100%).

Gelatin zymography was used to determine if deguelin inhibited migration and invasion was associated with inhibition of MMP-2 and -9 activities and data are presented in Figure 5A,B. Deguelin constitutively decreased MMP-2 and MMP-9 activities in a dose- and time-dependent manner.

### 2.5. Deguelin Alters Levels of Proteins Associated with Migration and Invasion in U-2 OS Cells

**Figure 6:**
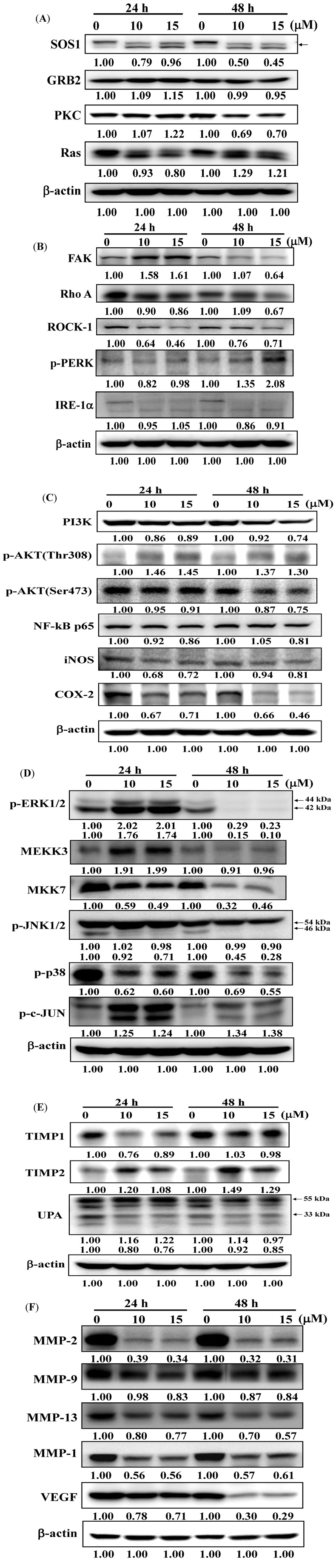
Deguelin affect the levels of associated proteins in migration and invasion of U-2 OS cells. U-2 OS cells were treated with deguelin (0, 10, 15 μM) for different periods of time and then cells were collected and the total protein extracts were prepared and determined as described in the Experimental Section. The levels of SOS1, GRB2, PKC and Ras (**A**), FAK, Rho A, ROCK-1, p-PERK and IRE-1α (**B**), PI3K, p-AKT(Thr308), p-AKT(Ser473), NF-κB p65, iNOS and COX-2 (**C**), MEKK3, MKK7, p-ERK1/2, p-JNK1/2, p-p38 and p-c-JUN (**D**), TIMP1, TIMP2, UPA (**E**), MMP-2, MMP-9, MMP-13, MMP-1 and VEGF (**F**) expressions were estimated by western blotting as described in the Experimental Section.

Deguelin effects several signal pathway, such as: cell growth and proliferation associated pathway (Figure 6A), cell motility and focal adhesion associated pathway (Figure 6B), PI3K/AKT/NF-κB pathway (Figure 6C), MAPK pathway (Figure 6D) and invasion associated pathway (Figure 6E,F). Levels of SOS1, GRB2, PKC and Ras (Figure 6A), FAK, Rho A, ROCK-1 and IRE-1α (Figure 6B), PI3K, p-AKT(Ser473), NF-κB p65, iNOS and COX-2 (Figure 6C), MEKK3, MKK7, p-ERK1/2, p-JNK1/2 and p-p38 (Figure 6D), UPA (Figure 6E), MMP-2, MMP-9, MMP-13, MMP-1 and VEGF (Figure 6F) were lower in deguelin-treated cells than that of control cells. Levels of p-PERK (Figure 6B), p-AKT(Thr308) (Figure 6C), p-c-Jun (Figure 6D), TIMP1 and TIMP2 (Figure 6E) were higher in deguelin-treated cells than that of control cells. GRB2 at 24 h treatment of deguelin was slightly higher than control but at 48 h treatment was lower than that of control (Figure 6A).

### 2.6. Effects of Deguelin on Protein Translocation

**Figure 7:**
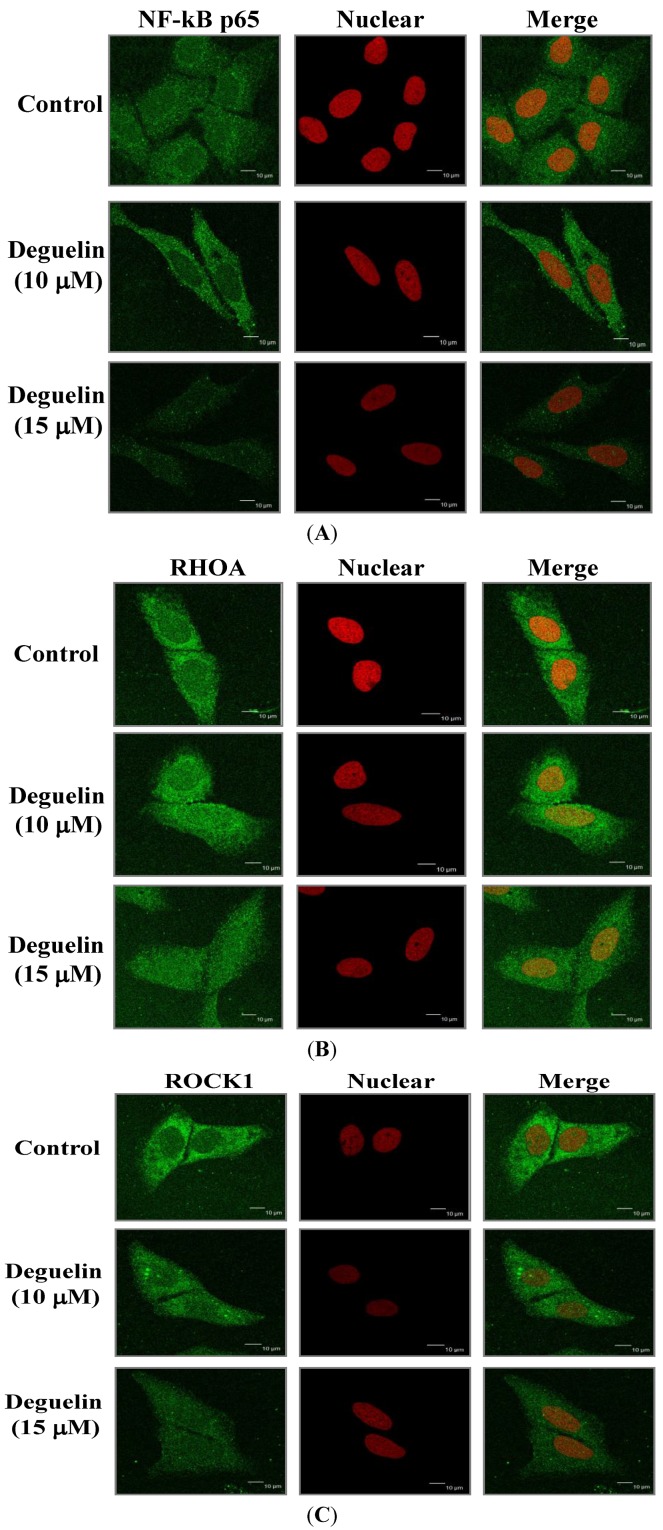
Deguelin affects the NF-κB p65, Rho A and ROCK-1 expression in U-2 OS cells. Cells placed on 6-well chamber slides were treated with 10, 15 μM of deguelin for 24 h, fixed and stained using anti-NF-κB p65 (**A**), Rho A (**B**) and ROCK-1 (**C**) antibodies (1:200) overnight and then stained with a secondary antibody (FITC-conjugated goat anti-mouse IgG at 1:200 dilution) (green fluorescence) followed by nuclear counterstaining individually performed with PI (red fluorescence). Photomicrographs were obtained using a Leica TCS SP2 confocal spectral microscope as described in the Experimental Section.

Expression levels of NF-κB p65, Rho A and ROCK-1 in U-2 OS cells were examined by immuno-staining and the results are shown in Figure 7A–C. Deguelin reduced cytosolic protein levels of NF-κB p65 (Figure 7A), Rho A (Figure 7B) and ROCK-1 (Figure 7C).

### 2.7. Effects of Deguelin on mRNA Expression of MMP-2, MMP-7, MMP-9, Rho A and NF-kB in U-2 OS Cells

**Figure 8:**
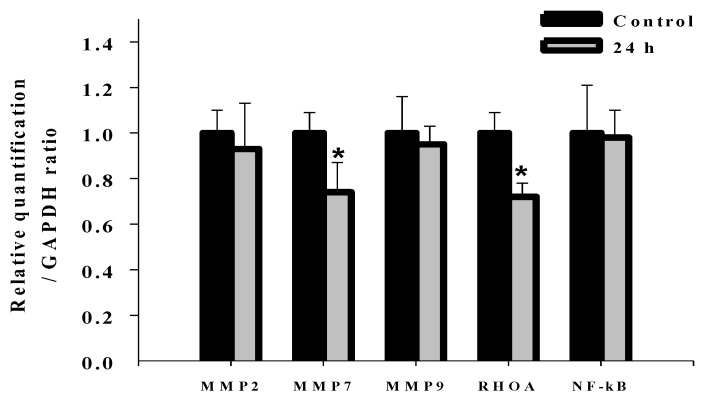
Real-time PCR of MMP-2, -7, and -9, Rho A and NF-κB. U-2 OS cells were incubated with deguelin (10 μM) for 24 h and then harvested for total RNA extraction for reverse-transcribed RNA samples at 42 °C with for 30 min. The revised RNA was used for quantitative PCR as described in the Experimental Section. Each assay were performed in triplicate and expression fold-changes were derived using the comparative C_T_ method. * *p* < 0.05, significant difference between deguelin-treated groups and untreated-groups as analyzed by Student’s *t* test.

In order to investigate whether deguelin affected migration- and invasion-associated gene expression in U-2 OS cells, cells were treated with deguelin (0 and 10 μM) for 0 and 24 h mRNA expression determined using real time PCR examination. Figure 8 shows that mRNA expression levels of MMP-7 and Rho A mRNA were significantly decreased by deguelin, but levels of MMP-2, MMP-9, NF-κB p65 mRNA were not significantly different.

### 2.8. Discussion

Numerous studies showed that deguelin has cytotoxic effects such as the induction of apoptosis by dysregulation of the cell-cycle checkpoint protein in retinoblastoma [25]. In our previous studies, we found deguelin induced DNA damage which was mediated by reducing DNA repair genes in human non-small cell lung cancer NCI-H460 cells [26]. Other reports have shown that deguelin has anti-cancer activity including the prevention of carcinogenesis in animal models of Sprague Dawley rats [17,18,19]. Cell motility plays an important role in cancer pathology, however, the effects of deguelin on cell motility are not well-understood. In the present study we found that deguelin reduced cell migration (Figure 2 and Figure 3) and invasion (Figure 4) of U-2 OS human osteosarcoma cancer cells *in vitro*. Effects of deguelin on cell motility were associated with inhibition of MMP-2 and -9 activities in a dose- and time-dependent manner (Figure 5A,B). MMP-2 and -9 are central players in cancer cell migration and invasion [27] and both have been implicated in cancer invasion [1,27].

Herein, we found deguelin reduced protein levels of MMP-2 and -9 and it also decreased the levels of FAK and the down-stream kinases ERK1/2, JNK and p38 in U-2 OS cells (Figure 6A,E,F). FAK/Src signaling was found to be involved in tumor metastasis by increasing cell migration and invasiveness [28,29]. Degulein inhibited the migration and invasion of U-2 OS cells may through the inhibition of FAK/src and ERK1/2. It was reported that FAK/Src may act on the down-stream targets PI-3K/AKt and Ras/Erk1/2 [30]. We showed that deguelin decreased protein levels of PI-3K, TIMP1, p-AKT (Ser473) in U-2 OS cells. It has been reported that deguelin acts as an Akt inhibitor by down-regulation of NF-κB signaling which induced apoptosis in colon cancer cells and inhibited tumor growth in mice [31].

We found that deguelin reduced NF-κB protein levels and decreased NF-κB levels in the cytosol of U-2 OS cells *in vitro*. It was reported that NF-κB can affect gene expression of MMP-2 and MMP-9 [32], herein, we suggesting deguelin inhibited MMP-2 and -9 activities which may occur by acting on ERK1/2MAPK and NF-κB signaling pathways. Deguelin inhibited the expression of growth factor receptor-bound protein 2 (GRB2), FAK and Rho A in U2-OS cells that were observed from western blotting (Figure 6). In the present study, the inhibition of GRB2, FAK and Rho A by deguelin may be due to a reduction in levels of MMP-2 and -9 and different lines of evidence support that hypothesis: (1) Rho A protein is associated with metastasis via cyclooxygenase-2 (COX-2) signaling promoting tumor cell motility [33]; and (2) the formation of FAK/Src complex allows Src to phosphorylate FAK and then to mediate its interaction with GRB2 and activate the Ras-ERK signaling pathway [34]. Other reports have demonstrated that angiogenic and metastatic signaling pathways acting through the RAS/RAF/MEK/ERK cascade are involved in the development of hepatocellular carcinoma cells and in the regulation of cell proliferation, apoptosis, cytokine expression and production of MMPs and VEGF in hepatocellular carcinoma (HCC) [35,36]. Deguelin was found to suppress pancreatic tumor growth and metastasis by inhibiting epithelial-to-mesenchymal transition in an orthotopic model [37]. Whether or not such a mechanism occurs in bone osteosarcoma warrants further investigations.

## 3. Experimental Section

### 3.1. Chemicals and Reagents

Deguelin, dimethyl sulfoxide (DMSO), propidium iodide (PI), Tris-HCl Trypsin, and Trypan blue were obtained from Sigma Chemical Co. (St. Louis, MO, USA). McCoy’s 5A medium with L-glutamine, fetal bovine serum, penicillin-streptomycin, and trypsin-EDTA were obtained from Gibco BRL (Grand Island, NY, USA). All chemicals and reagents were of analytical grade. 

### 3.2. U-2 OS Cell Culture

The U-2 OS human osteosarcoma cell line was obtained from the Food Industry Research and Development Institute (Hsinchu, Taiwan) and kept frozen under liquid nitrogen in 10% DMSO with fetal bovine serum until use. U-2 OS cells were quickly thawed and maintained in 90% McCoy’s 5A medium with 10% FBS, 2 mM l-glutamine, 100 Units/mL penicillin and 100 μg/mL streptomycin and plated onto 75 cm^2^ tissue culture flasks at 37 °C under a humidified 5% CO_2_ atmosphere as described previously [38,39].

### 3.3. Cell Viability Assay

U-2 OS cells were seeded onto 12-well plates at a density of 2 × 10^5^ cells/well and treated with deguelin (0, 5, 10, 15 and 20 μM) or 0.5% DMSO (as a vehicle control) for 24 and 48 h. At the end of the incubation period, cells were harvested and stained with 5 μg/mL of PI and then were analyzed using a PI exclusion method by flow cytometry (BD Biosciences, FACS Calibur, San Jose, CA, USA) as previously described [38,39].

### 3.4. Scratch Wound Healing Assay

U-2 OS cells at a density of 5 × 10^5^ cells/well were cultured in 6-well plates with serum-free McCoy’s 5A medium until they were 100% confluent in an adherent monolayer. A sterile yellow 10 μL Eppendorf tip was used to scratch the cells in the plate and then washed with PBS three times. The cells were then placed in fresh serum-free McCoy’s 5A medium containing 0, 10 and 15 μM of deguelin for 0, 12, 24 h then random fields were examined, selected and photographed with an inverted microscope as described previously [40,41]. 

### 3.5. Cell Invasion and Migration Assays in Vitro

Cell migration and invasion assays were conducted using the Matrigel Cell Migration Assay and Invasion System as described previously [42,43]. The Migration assay for cell migration was conducted using transwell (BD Biosciences, Franklin Lakes, NJ, USA) cell culture chambers (8 mm pore size; Millipore, Billerica, MA, USA). U-2 OS cells were maintained in serum-free McCoy’s 5A medium for 24 h. Cells were then trypsinized and resuspended in serum-free McCoy’s 5A medium, and cells at 5 × 10^4^ cells/well were placed in the upper chamber of the transwell insert and incubated with 0.5% DMSO or deguelin (10 and 15 μM), and 90% McCoy’s 5A medium containing 10% FBS was added to the lower chamber and incubated for 24 or 48 h. In the upper chamber, cells remaining were removed by wiping with a sterile cotton swab and in the lower surface of the filter, the migrated cells were fixed with 4% formaldehyde in PBS and stained with 2% crystal violet in 2% ethanol and then were counted and photographed with a light microscope at 200×. The cell invasion assay was performed as described except that the filter membrane was coated with Matrigel from a BioCoat Matrigel invasion kit (Becton Dickinson Bioscience, Bedford, MA, USA). Cells located on the underside of the filter were counted and photographed with a light microscope at 200×. The invasive cells were expressed as the mean number of cells (mean ± SD of cells) on the lower side of the filter. The assay was done in triplicate. The experiment was repeated three times. 

### 3.6. Gelatin Gel Zymographic Assay for MMP Activity

U-2 OS cells at a density of 5 × 10^5^ cells/well were plated in 12-well plates for 24 h. Deguelin (0, 2.5, 5, 10, 15 and 20 μM) was then added to well and cells were then incubated at 37 °C for 24 and 48 h. At the end of the incubation periods, the supernatant from each concentrated culture was re-suspended in non reducing loading buffer and incubated at 37 °C for 15 min. Each sample was prepared in 10% SDS-PAGE cast with 0.1% gelatin and then electrophoresed. After electrophoresis, gels were washed successively with 50 mL of 2.5% (v/v) Triton X-100 in distilled water twice (30 min each) and 2.5% (v/v) Triton X-100 in Tris buffer twice (30 min each) to remove SDS. Gels were then incubated in a developing buffer (50 mM Tris-HCl (pH 7.8) 10 mM CaCl_2_, 150 mM NaCl) for 16 h at 37 °C. Gels were then stained with Coomassie Brilliant Blue R 250 and destained with 30% methanol, 10% acetic acid in order to reveal zones of substrate lysis for detecting gelatinase secretion [44]. In the blue backgroung gel, the gelatinolytic activity was visualized as clear zones and the intensity was measured using Quantity One software as described previously [44].

### 3.7. Immunofluorescence Staining and Confocal Laser Scanning Microscopy

U-2 OS cells at a density of 5 × 10^5^ cells/well were plated on 4-well chamber slides before being treated with 0, 10 and 15 μM of deguelin for 24 h. Cells were then fixed in 3% formaldehyde in PBS for 15 min. Triton X-100 (0.1%) in PBS was used to permeabilize cells for 1 h with blocking of U-2 OS cells at a density of 5 × 10^5^ cells/well and were placed in n-specific binding sites using 2% BSA as described previously (A, B). After the fixation, cells on the slides were stained with anti-NF-kB, Rho A and ROCK1 (1:200 dilution) as primary antibodies overnight and then were stained with FITC-conjugated goat anti-mouse IgG at 1:200 dilution) (green fluorescence) as a secondary antibody. The mitochondria and nuclei were counterstained individually with MitoTracker^®^ Red CMXRos and PI (Molecular Probes/Invitrogen Corp., Carlsbad, CA, USA) (red fluorescence). All samples were photomicrographed with a Leica TCS SP2 confocal spectral microscope as described previously [27,28].

### 3.8. Western Blotting Analysis

U-2 OS cells at a density of 1 × 10^6^ cells/well were plated on 6-well plates for 24 h. Deguelin (10 and 15 μM) was added to wells while DMSO (solvent control) was added to control cells and incubated at 37 °C for 24 and 48 h. Cells were then harvested and lysed with ice-cold 50 mM potassium phosphate buffer (pH 7.4) containing 2 mM EDTA and 0.1% Triton X-100. Cells were then homogenized in a Dounce homogenizer with optimal gentle strokes followed by centrifugation at 13,000 rpm for 10 min at 4 °C. The supernatant was collected as the cytosolic proteins and total protein was determined using a Bio-Rad protein assay kit (Hercules, CA, USA) with bovine serum albumin (BSA) as the standard. Equal amounts of proteins from each sample were separated on 10% SDS-polyacrylamide gels, followed by electrophoretic transfer to PVDF (polyvinylidene fluoride, Millipore, Bedford, MA, USA) membranes. The membranes were blotted with the relevant primary antibodies, washed and then were stained with a secondary antibody and protein bands were detected with an enhanced chemiluminescent substrate (ECLTM, Amersham Biosciences, Amersham, UK). Bands were quantified using NIH Image analyzer software (NIH, Bethesda, MD, USA) [42,44].

### 3.9. Real-time PCR of MMP-2, -7, and -9, Rho A and NF-kB

U2-OS cells (1 × 10^6^ cells/well) were plated in 6-well plates for 24 h and then were incubated with deguelin (10 μM) for 24 h and then harvested for total RNA extraction using the Qiagen RNeasy Mini Kit as described previously [18,19,20,21]. High Capacity cDNA Reverse Transcription Kit was used for reverse-transcribed RNA samples at 42 °C for 30 min according to the protocol of the supplier (Applied Biosystems, Carlsbad, CA, USA). The revised cDNA was used for quantitative PCR and the conditions were: 2 min at 50 °C, 10 min at 95 °C, and 40 cycles of 15 s at 95 °C; 1 min at 60 °C using 10 ng/μL of the cDNA reverse-transcribed as described above, 2X SYBR Green PCR Master Mix (Applied Biosystems) and 200 nM of forward and reverse primers as shown in Table 1 as described previously [18,19,20,21]. Each assay were performed using an Applied Biosystems 7300 Real-Time PCR system in triplicate and expression fold-changes were calculated using the comparative C_T_ method [45,46,47,48].

**Table 1:** The DNA sequence was evaluated using the Primer Expression.

Primer Name	Primer Sequence
homo MMP-2-F	CCCCAGACAGGTGATCTTGAC
homo MMP-2-R	GCTTGCGAGGGAAGAAGTTG
homo MMP-7-F	GGATGGTAGCAGTCTAGGGATTAACT
homo MMP-7-R	AGGTTGGATACATCACTGCATTAGG
homo MMP-9-F	CGCTGGGCTTAGATCATTCC
homo MMP-9-R	AGGTTGGATACATCACTGCATTAGG
homo NF-κB-F	AGTTGAGGGGACTTTCCCAGGC
homo NF-κB-R	TCAACTCCCCTGAAAGGGTCCG
homo RhoA-F	TCAAGCCGGAGGTCAACAAC
homo RhoA-R	ACGAGCTGCCCATAGCAGAA
Homo GAPDH-F	ACACCCACTCCTCCACCTTT
Homo GAPDH-R	TAGCCAAATTCGTTGTCATACC

MMP, Matrix metalloproteinase; NF-κB, nuclear factor-kappaB; RhoA, RAS homologue gene family member A; ROCK1, Rho-associated coiled coil-containing kinase; GAPDH, Glyceraldehyde-3-phosphate dehydrogenase; F, forward primers; R, reverse primers.

### 3.10. Statistical Analysis

All results were done in 3 independent experiments and expressed as mean ± SD. Differences between the deguelin-treated and control groups were analyzed by Student’s *t* test, with values of * *p* < 0.05 considered significant.

## 4. Conclusions

**Figure 9:**
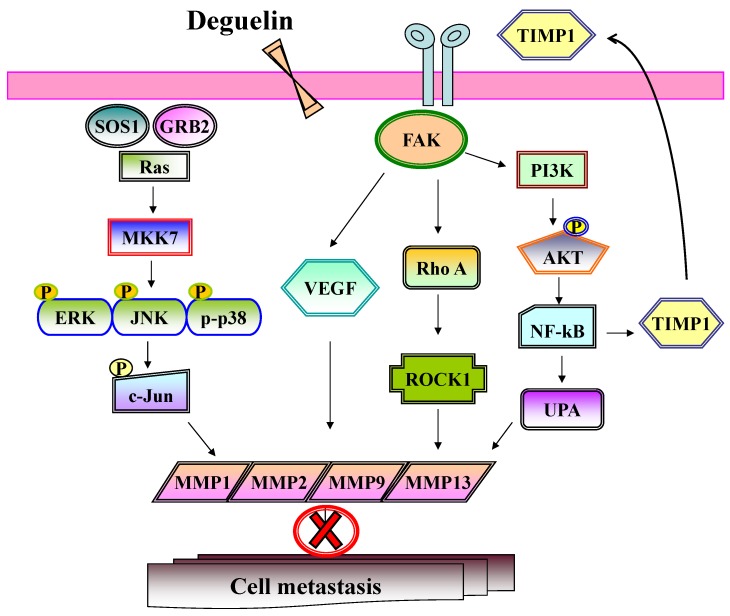
The possible signaling pathways for deguelin inhibited cell invasion and migration in U-2 OS human osteosacroma cells.

In conclusion, we show for the first time that deguelin inhibits cancer cell migration and invasion in U-2 OS cancer cells. Several different signaling pathways may be involved in these inhibitor actions which are depicted in Figure 9. Numerous studies have shown deguelin induced cytotoxic effects in human cancer cells [26,31,49,50]. Herein, we propose that deguelin may have potential as a novel anti-cancer agent for the treatment of osteosarcoma by inhibition of migration and invasion and provides the rationale for *in vivo* studies in animal models.
